# A Functionalistic Stress Recovery Intervention Improves Perceived Recovery Opportunities and Relaxational Behaviors: A Secondary Analysis of a Randomized Controlled Trial

**DOI:** 10.3390/ijerph192114005

**Published:** 2022-10-27

**Authors:** Niclas Almén

**Affiliations:** Department of Psychology and Social Work, Mid Sweden University, 83125 Östersund, Sweden; niclas.almen@miun.se

**Keywords:** stress management: behavior change, intervention, prolonged stress, stress recovery, recovery experiences, control, relaxation, mastery, RCT

## Abstract

The recovery perspective on stress management is new and few recovery intervention studies have been conducted. The aim of the study was to evaluate behavioral effects of a functionalistic stress recovery intervention, in which individuals perceiving high levels of stress were encouraged to pay attention to possibilities to perform potential recovery behaviors in everyday life and to choose behaviors that were predicted to lead to resource restoration. Seventy-three individuals were randomly allocated to either a 10-week intervention or a waiting-list control group. Three types of recovery behavior factors during leisure time were studied: perceived recovery opportunities (i.e., control), relaxational behaviors (i.e., relaxation), and positively challenging behaviors (i.e., mastery). In comparison with the control group, the intervention group significantly improved, showing high between-group effect sizes, regarding perceived recovery opportunities (*p* < 0.001; *d* = 0.75) and relaxational behaviors (*p* < 0.001; *d* = 0.80). Both groups normalized their levels of positively challenging behaviors between pre- and postassessment, and no statistically significant group difference was demonstrated. Analyses of reliable and clinically significant changes demonstrated results in favor of the intervention group regarding perceived recovery opportunities and relaxational behaviors but not positively challenging behaviors. The tested intervention warrants further research, for example, if a modified version of the intervention including components aiming at increasing postwork positively challenging behaviors would be beneficial for the improvement of the behavior and for health.

## 1. Introduction

Modern life includes repeated exposure to situations in which stress responses are activated [1]. These are important for handling threatening situations [2]. They can also enhance “positive” emotions [3] and performances [4]. However, *prolonged* stress responses are harmful, possibly resulting in low levels of wellbeing and ill health, such as exhaustion/burnout [2,5,6]. In particular, work stress has been shown to be associated with prolonged stress-related ill health [7]. There is moderate-quality evidence that work stressors in terms of effort–reward imbalance, low procedural and relational justice, high job demands, low coworker and supervisor support, high emotional demands, and low decision authority increase the incidence of stress-related disorders from 20 to 90 percent [7].

Prolonged stress responses imply a lack of stress recovery (henceforth usually only named recovery). Accordingly, several researchers have explained stress-related ill health, such as burnout, explicitly in terms of impaired recovery processes after periods of stress and strain [8,9,10]. Although recovery at work is likely to be of importance [11,12], there is the greatest evidence that recovery in leisure time is highly important [13]. It seems possible to help people at risk for developing stress-related ill health, such as burnout, by strengthening their recovery-facilitating behaviors after stressful situations and effort expenditure [14]. However, the recovery perspective on stress management is new and fairly unexplored. Skinner et al. [15] analyzed 100 assessments of coping, which included 400 ways of coping, and recovery or associated concepts such as restoration, recuperation, or deactivation was not included even once. I suggest considering recovery behaviors in poststress situations as a stress-coping domain, which needs to be studied as such. There are a lot to learn, for example, how stress management interventions affect the individual’s recovery-coping behaviors.

Recovery can be facilitated through different types of behavior. It is possible to distinguish between behaviors characterized by low levels of activation, demands, effort, and challenges, and behaviors characterized by a moderate and positive challenge, some demands, learning, and experiences of mastery [16,17]. I label the first type as *relaxational behaviors* and the latter as *positively challenging behaviors*. Relaxation can be accomplished using a relaxation technique or by an activity that requires low levels effort, such as reading a novel, watching a TV show, listening to music, and walking in the woods, whereas reading a scientific article, playing a sport, learning carpentry, and meeting new people are examples of activities that may involve a positively experienced challenge. Relaxational behaviors are clearly related to the definition of a stress recovery process as psychophysiological deactivation after stress and effort expenditure [18]. Although positively experienced challenging behaviors are not that clearly associated to that process, it prospectively predicts recovery outcomes such as decreased levels of exhaustion/burnout [19]. Mentally detaching from stress in the poststress situation is important for recovery [20]. Positively challenging behaviors, which generally may require more mental attention than relaxational behaviors, can be helpful for such detachment. Importantly, potential positively experienced challenging behaviors, such as physical exercising, may be accompanied by physical stress responses and a subsequent enhanced physical recovery process. For example, if you exercise before unwinding on the couch after a stressful day at a sedentary job, the level of relaxation has a greater potential of being high than if you immediately unwind on the couch [21]. Accordingly, positively challenging behaviors may sometimes not be a recovery behavior in itself but a facilitator of the effectiveness of subsequential recovery behaviors. Importantly, during an activity, the individual may experience both relaxation and challenges. For example, for some people with high levels of stress, practicing a relaxational method is a challenging task. The correlation between positively challenging behaviors and relaxational behaviors during leisure time usually varies between 0.3 [17] and 0.6 [22]. A meta-analytical examination of the (corrected) correlations between these two factors and fatigue indicated that relaxational behaviors have a stronger relationship (−0.35) with fatigue than positively challenging behaviors (−0.18) [16]. A study by Trogólo et al., (2020) demonstrated an almost identical relationship between the two types of behaviors and burnout/exhaustion (−0.39 and −0.20, respectively) [23].

Before a person decides which behavior to engage in to recover from stress, there is a need to have, as well as perceive having, room for a recovery-facilitating behavior. In line with this, to experience that you can decide for yourself what to do in your free time is associated with recovery [13,22], and persons perceiving high levels of demands and stress may not have or perceive this opportunity [17]. Bennet et al., (2016) compared different ways of recovering during leisure time among employees, and the combination of fairly high levels of perceptions of recovery opportunities, relaxational behaviors, positively challenging behaviors, and constructive reflection on work challenges, which is the most common, indicated a preference for these behaviors [24]. In addition, from an outcome point of view, this “recovery profile” was the most desirable as it was associated partly with well-being (indicated by somatic complaints at low levels of exhaustion) and partly with work engagement.

Only a few studies have investigated interventions that directly aimed to intervene both positively challenging behaviors and relaxational behaviors, and the results showed that improvements can be achieved. For example, in a study by Hahn et al., (2011), such an intervention led to roughly equal effects for the two types of behaviors (relaxational behaviors: *d* = 0.58, and positively challenging behaviors: *d* = 0.68) [25]. It is unclear what effects can be achieved via a recovery behavior intervention where no specific types of recovery behaviors are intervened but where the choice of type of behavior change is based on the person’s own self-observations, preferences, and predictions.

The aim of the present study was to investigate whether subjects with high levels of perceived stress participating in a functionalistic stress recovery intervention, in which individuals were encouraged to pay attention to possibilities to perform potential recovery behaviors and to choose behaviors that were predicted to lead to high levels of recovery outcomes in poststress situations in everyday life, in comparison with a waiting-list control group, would increase perceptions of recovery opportunities, relaxational behaviors, and positively challenging behaviors, during off-job time.

## 2. Materials and Methods

### 2.1. Inclusion and Exclusion Criteria

The inclusion criteria were (a) experiencing stress symptoms (such as difficulty to relax, tiredness, restlessness, headache, tension, irritability, worry, and difficulty in concentrating); (b) high levels of perceived stress (≤25 on the Perceived Stress Scale, PSS); (c) professional work at least 20 h per week, (d) being aged 25 to 55; (e) having practical possibilities of participating in the intervention (i.e., daily access to a computer with internet access, possibilities of participating in the live meetings, and ability to communicate in Swedish). The exclusion criteria were problems or circumstances that were expected to complicate participation in the program, such as substance abuse and severe psychiatric problems, and/or indicated that other interventions were more relevant and, therefore, should be prioritized.

### 2.2. Design

Participants were recruited through advertisements in the local newspapers (Östersund, Sweden), on Facebook, and on Google. All individuals fulfilling the criteria were randomized to either an intervention group (INT) or a waiting-list control group (WLC). An intention-to-treat approach was used [26]. Allocation to either the INT or the WLC was conducted consecutively, in five waves. A block randomization was used and out of 219 individuals who signed up for the study, 73 were randomized to either the INT (*n* = 35) or the WLC (*n* = 38). Two intervention completers and seven out of the eleven participants who did not complete the intervention withdrew from the postassessment. From the WLC-group, five did not complete the postassessment.

### 2.3. Assessment and Measures

The PSS (14 items; scale: 0–4) measures the degree to which situations in a person’s life are appraised as stressful [27]. Eskin and Parr [28] have reported a PSS mean value of 24.4 using a sample of university students in Sweden. The Cronbach’s alpha value is often around 0.8 [27,28]. In the present sample, Cronbach’s alpha was 0.74. Data from the self-report outcome measures used in the present study were collected (online) immediately before (pre) and immediately after the intervention (post). The Swedish versions of the following self-report measure were used as outcome measures: Control (four items), Relaxation (three items), and Mastery (four items), all subfactors included in the Swedish version of the Recovery Experiences Questionnaire (scale 1–5) measuring experiences associated with postwork recovery [22]. All three factors are related to several well-being indicators [17]. The Swedish version of the REQ has been proven to have good convergent and discriminant validity for all factors [22]. The composite reliabilities in a general Swedish sample were 0.89 for Control, 0.87 for Relaxation, and 0.84 for Mastery. The Control subscale was used as an indicator of *perceived opportunities to engage in recovery behaviors during off-job time*. The items were: ”I feel like I can decide for myself what to do”, ”I decide my own schedule”, ”I determine for myself how I will spend my time”, and ”I take care of things the way that I want them done”. Cronbach’s alpha in the present sample was 0.88. The subscale Relaxation was used as an indicator of *relaxational behaviors during off-job time*. The items were: ”I kick back and relax”, ”I use the time to relax”, and ”I take time for leisure”. Cronbach’s alpha in the present sample was 0.88. The subscale Mastery was used in order to measure *positively challenging behaviors during off-job time*. The scale includes the following items: ”I learn new things”, ”I seek out intellectual challenges”, ”I do things that challenge me”, and ”I do something to broaden my horizons”. In the present sample, Cronbach’s alpha was 0.78.

### 2.4. Background Data

Background data were collected via questions online at preassessment. Among the total of 73 participants who entered the study (of which 55 were women), the mean age was 41.4 years (SD 7.43). Work positions were distributed as follows: high-level managers (*n* = 6), low-level managers (*n* = 6), blue-collar workers (*n* = 21), white-collar workers (*n* = 17), temporarily employed (*n* = 13), and others (*n* = 9). Around half (*n* = 35) of them worked approximately 40 h per week (which is the general norm in Sweden). Twenty-five participants worked more and 12 worked less than that. The participants attributed stress symptoms to a larger extent to the working situation than their private life. Only six participants did not agree at all that their stress symptoms were due to their private life. Sixty-five participants had no sick-leave and the mean of the level of sick-leave for the remaining 6 participants was 75 percent of (data were missing from two participants). The mean score on the PSS was 33.32 (SD 5.64) and that on the burnout score measured by the Shirom–Melamed Burnout Questionnaire was 4.78 (SD 0.82), which is in between the 75th and 90th percentiles in a general Swedish population [29], and 0.46 standard deviations above the cut-off score (4.4) for severe burnout suggested by Lundgren-Nilsson et al. [30]. The mean scores of Control, Relaxation, and Mastery were 0.69, 0.66, and 0.59 standard deviations lower, respectively, than the mean values of a Swedish general population [31].

### 2.5. The Intervention

The intervention used in the present study, BRIGHT-recovery (BRIGHT is an acronym for Behavioral Recovery Intervention for General Health and Thriving; in recent studies, the intervention has been labeled ”Balance in everyday life”), has been tested in several studies [8,14,32]. In the previous study of the intervention, assumed effects of recovery behaviors, but not such behaviors, were studied, and at postintervention, all measures (burnout, the burnout antecedents perceived stress and tension, and the burnout concomitants anxiety and depression) demonstrated high between-group-effect-sized improvements (Cohen’s *d* ≥ 0.8) [33]. In the present study, the same sample was used.

The intervention consisted of seven 2.5 h group sessions including 3–8 participants over a period of approximately 10 weeks, led by me, who is a licensed psychologist and licensed psychotherapist with training in cognitive–behavioral therapy. The primary targets in the intervention were to engage in any type of behavior (at work and outside of work) that were possible to perform and predicted to lead to recovery effects (such as restored levels of vitality), in the current situation. The general goal was to use five recovery potential behaviors every day with a minimum duration of ten minutes, and to evaluate the recovery outcomes of the specific behavior in the specific situation in which the behavior was used. The participants were trained in the coping technique Applied tension release [34] and encouraged to use the skill during recovery behaviors, for example, when watching TV or reading or riding a bike, in order to enhance the psychophysiological deactivation (i.e., the recovery processes) and the recovery effects (e.g., vitality). A range of intervention components were used to support what has been described above, such as psychoeducation and rationale for the change in recovery behaviors, behavior planning, behavior diaries, modeling, recovery training during the sessions, positive feedback, and identification of recovery supporting factors and recovery obstacles in everyday life.

The most innovative aspects of this intervention was the emphasis that recovery at work was generally as important as recovery during leisure time; that the participant themselves had to select, test, and evaluate the recovery outcomes of different activities in different situations, to use a general frequency goal of five recovery-potential activities per day, and to perform the training of relaxation skills with the (sole) purpose of strengthening psychophysiological deactivation in recovery situations; that the therapist completely refrained from supporting other forms of change (e.g., stress reduction when exposed to external stressors) than improved recovery behaviors. For a more detailed description of the intervention, see Almén et al., (2020) [32].

### 2.6. Statistical Analysis

To check for comparability between groups at pretreatment, a series of independent group *t* tests and chi-square tests were conducted.

One participant (in the INT) did not complete the assessment of the outcome variables at pretreatment, and fourteen participants (nine in the INT and five in the WLC) did not complete the posttreatment assessment (i.e., 10.27 percent of the assessments were missing). The amount of missing outcome variable data on item level was 10.46 percent. Missing data were imputed at item level for each condition separately using multiple imputation (MI), creating 10 imputed datasets [35,36]. The random number generator, Mersenne Twister, was used. The model type used was predictive mean matching with the fully conditional specification. To minimize overfitting, the number of predictors was limited [37,38] to four: age and the outcome measures (Control, Relaxation, and Mastery), assessed at pretreatment. The imputation was based on the assumption of missing data at random [39,40,41]. A series of one-way analysis of covariance (ANCOVA) were performed to determine statistically significant differences at posttreatment between the INT and the WLC on each outcome variable, controlling for the pretreatment assessment scores on the variable analyzed [42]. The results from these analyses were pooled using Rubin’s rules [43]. Between-group effect sizes at posttreatment were calculated using Cohen’s *d;* (M_INT_–M_WLC,_ with adjustments for preassessment values)/SD_pooled_. In order to determine the level of effects, the commonly used suggestions by Cohen (1988) were used: small (*d* = 0.2), medium (*d* = 0.5), and large (*d* = 0.8) [33].

In addition, analyses of reliable and clinically significant changes regarding Control, Relaxation, and Mastery at posttreatment using the recommendations by Jacobson and Truax (1991) were conducted [44]. Among the three alternative formulas for calculating cutoff scores, I chose the criteria consisting of the value that lies within the weighted midpoint between the norm group and participants’ mean defined as: [(*SD*_participant_ × mean_norm group_) + (*SD*_norm group_ × mean_participants_)]/(*SD*_participant_ + *SD*_norm group_), yielding ≥3.06 points for Control, ≥2.82 points for Relaxation, and ≥2.57 points for Mastery. Using the formula of Jacobson and Truax (1991), Reliable Change Index was calculated to be at least a 0.91-point reduction on Control, a 0.99-point reduction on Relaxation, and a 0.89-point reduction on Mastery. Based on the Reliable Change Index, the result for the participants on each measure at post- and follow-up assessment was categorized as a *reliable improvement*, a *no change*, or a *reliable deterioration*. If the cutoff score criterion was met among those categorized as reliably improved, a *clinically significant change* had been accomplished [44]. Fisher’s exact probability test (2-tailed) was used to analyze differences between groups.

For further information regarding the research methodology, such as recruitment, descriptions of the procedure, sample characteristic, the intervention, and/or reasons for dropping out, see Almén et al. [32]. All statistical analyses were performed using JASP, version 0.13.0, for Mac (released 2020 by JASP Team) except for the multiple imputation that was performed using IBM SPSS Statistics for Mac, version 27.0 (released 2020 by IBM Corp., Armonk, NY, USA).

## 3. Results

### 3.1. Pretreatment Comparisons

No significant differences on any background (such as age, gender, perceived physical health, attribution to stress symptoms, working and conditions, and sick leave) or outcome variables were found between the INT (*n* = 35) and the WLC (*n* = 38), or between participants that completed (*n* = 59) and did not complete (*n* = 14) the postassessment.

### 3.2. ANCOVAs

After adjusting for the scores on the Control-scale at pretreatment assessment, there were statistically significantly higher (i.e., better) scores on the Control in the INT compared with the WLC at postassessment, *t*(1, 70) = 3.95, *p* < 0.001. After adjusting for the scores on the Relaxation-scale at pretreatment assessment, there were statistically significantly higher (i.e., better) scores on the Relaxation in the INT compared with the WLC at postintervention assessment, *t*(1, 70) = 3.78, *p* < 0.001. After adjusting for the scores on the Mastery-scale at pretreatment assessment, there were no statistically significant differences on the Mastery between the INT and the WLC at posttreatment assessment, *t*(1, 70) = 0.76, *p* = 0.446.

Means and standard deviations for the outcome variables at pre- and postassessments for each group (INT and WLC) are presented in Table 1. In addition, differences in means between the groups at postassessments and *p* values from ANCOVAs with adjustments for preassessment values, with 95% CI, and between-group effect sizes (Cohen’s *d*), are presented in the table.

### 3.3. Reliable and Clinically Significant Changes

Table 2 displays how many participants from the INT and the WLC ended up in each outcome category on the measures: Control, Relaxation, and Mastery, at posttreatment. The INT (compared to the WLC) had a significantly higher proportion of reliable improvements (*p* = 0.002) as well as clinically significant changes (*p* = 0.005) regarding Control, and the WLC had a significantly higher proportion of no change (*p* = 0.028). No statistically significant differences between the groups regarding reliable improvements or no change were demonstrated on Relaxation. However, statistically significant differences regarding clinically significant improvements (*p* = 0.010) and reliable deterioration (*p* = 0.026) in favor of the INT were demonstrated. When it comes to Mastery, no statistically significant differences between the groups were shown for any of the four outcome categories.

## 4. Discussion

To the best of my knowledge, this is the first randomized controlled trial investigating the effects of a functionalistic stress recovery intervention—in which individuals were encouraged to perform self-chosen behaviors that were recovery-functional at the specific time they were performed—on perceptions of recovery opportunities, relaxational behaviors (which is characterized by low levels of activation, demands, effort, and challenges), and positively challenging behaviors (characterized by a moderate and positive challenge, some demands, learning, and experiences of mastery) during off-job time among people with high levels of perceived stress.

Research shows that recovery-facilitating behaviors become less likely when the number of stressors is high and recovery is needed [16,45]. This study supports, at least partially, the assumption that an intervention aiming at strengthening recovery behaviors in everyday life may be a way to solve this recovery paradox. In comparison with the WLC, the INT improved perceived recovery opportunities and relaxational behaviors but not positively challenging behaviors off work. Below, I reflect on the results for each outcome variable.

*Perceived recovery opportunities* (during leisure time). A possible common reason for the recovery paradox is that when we are experiencing prolonged stress and a need for recovery, we lack experiences of opportunities for recovery. The intervention led to statistically significant and high-level-effect-sized improvements in comparison with the WLC. In addition, the intervention led to a higher proportion of reliably improved and clinically significant improved participants compared to the WLC. The mean value on the Control scale in a Swedish general population sample was 3.41 [31], which was exactly the score for the INT at postassessment. The intervention may have intervened perceptions of recovery opportunities by continuously supporting the individual to plan for recovery behaviors, perhaps implicitly signaling that almost everyone has, or can create, recovery opportunities. If a person is repeatedly asked to plan for recovery behaviors, they may increase the perceptions of opportunities that were not previously perceived. Perceptions of recovery opportunities may also increase as a function of experiences of having practiced a recovery behavior and positive experiences of the behavior. Consequently, recovery behavior and perceptions of recovery opportunities may mutually influence each other. Notably, the intervention did not aim to support the removal of any other activity or stressor in order to receive room for recovery. Stressors in life were normalized. The improvements in recovery opportunities may be of primary importance for solving the recovery paradox.

*Relaxational behaviors* (during leisure time). The INT increased relaxational behaviors, in comparison with the WLC, and the in-between-group effect size was high. Statistically significant differences regarding clinically significant improvements (*p* = 0.010) and reliable deterioration (*p* = 0.026) in favor of the INT were demonstrated. The mean value of the Relaxation scale in a Swedish general population sample was 3.19 [31], and the INT surpassed that score at postassessment. This improvement is very promising as relaxational behaviors during leisure time are clearly related to the essence of the proposed process leading to recovery: psychophysiological deactivation after stress and effort. Notwithstanding, research demonstrates mixed results regarding relaxational and low-effort activities effects on recovery from stress. For example, whereas Bennet and Bakker (2018) [16], in their meta-analysis of recovery experiences, showed that recovery experiences are important for fatigue and vigor, a study by Rock and Ziljstra (2006) [46] indicated that low-effort activities were not helpful for recovery to occur, as they were associated with increased levels of fatigue. These seemingly inconsistent findings may be explained by the fact that low-effort activities do not necessarily mean that the person is relaxed. Low-effort activities can be associated with rumination and depression [47], which includes stress rather than recovery responses [48,49]. This supports the assumption that it is important to pay attention to how people behave covertly, i.e., whether they deactivate or not when performing various activities in potential recovery situations and offer help to those who have difficulty relaxing in these situations to do so (for example, via tension-release skills training).

*Positively challenging behaviors* (during leisure time). The INT did not statistically significantly increase positively challenging behaviors, in comparison with the WLC. Accordingly, the analyses of reliable and clinically significant changes did not show any differences between the groups. The lack of between-group differences seems to, in part, be due to the fact that not only the INT but also the WLC made statistically significant (paired samples *t*-tests; *p* < 0.001) improvements between pre- and postintervention, and with Cohens’s *d* values of 0.64 for the INT and 0.45 for the WLC, suggesting approximately medium-level within-group effect sizes. Both groups reached approximately the mean value (2.91) of the Mastery scale in a Swedish general population sample [31]. The WLC did not change the levels of relaxational behavior or perceived recovery opportunities between pre- and postintervention according to the conducted paired samples *t*-tests.

The result of the study supports the assumption that the studied recovery factors are state and not trait factors, probably controlled by a range of factors that we need to learn more about.

Although it was not part of the purpose of the study, it may be of interest to report results from some prediction analyses: According to linear regression analyses based on change scores for the total sample using the measure Adjusted R square, improved perceived recovery opportunities explained 17.1 percent (*p* < 0.001) of the variance in relaxational behaviors, and 12.8 percent (*p* = 0.002) of the variance in positively challenging behaviors. Relaxational behaviors and challenging behaviors explained 12.4 percent (*p* = 0.002) and 11.0 percent (*p* = 0.003), respectively, of the reduction in Burnout at post (burnout effects of the intervention have been reported earlier, see Almén et al. [32]). Changes in perceived recovery opportunities explained 8.1 percent (*p* = 0.009) of the changes in burnout. Together, relaxational behaviors and positively challenging behaviors accounted for 18.2 percent (*p* < 0.001) of the reduction, indicating that it is of importance to change both behaviors in order to reduce burnout. Adding perceived recovery opportunities to the explanatory model, the model independent variables explained 18.0 percent (*p* = 0.001) of the burnout reduction, which is in line with, but does not prove the correctness of, the assumption in the present paper that the measure control covers perceived recovery opportunities and not recovery behaviors.

### 4.1. Limitations

The study has some limitations. First, although the recommended multiple imputation method was used [39,50], the amount of missing data (10.46%) could influence the conclusions that were drawn from the data. Second, the intervention methodology could have been confounding with the therapist factor as only one therapist was used. Third, therapist behavior was not assessed, which means that it was impossible to analyze/assess if the therapist supported or undermined the studied variables. Fourth, concomitant interventions or other factors possibly influencing the studied variables were not controlled for. Therefore, it was not possible to find plausible causes of the improvements in positively challenging behavior between pre- and postintervention in the WLC. Fifth, as the variables were only assessed pre- and postintervention, it was not possible to analyze perceived recovery opportunities as a mediator of the change in relaxational and challenging behaviors.

### 4.2. Future Research

The result of this study warrants investigation of whether the type of intervention tested in the present study, in different settings, using different therapists, and including different populations, will lead to the same or different effects that have been demonstrated in the present study. Analyses of reliable and clinically significant changes show that there are many individuals who do not reliably improve the studied behaviors, which motivates investigating the reason for the change in behavior of some but not others. It would also be valuable to study if a modified version of the intervention including components aiming at eliciting and reinforcing postwork positively challenging behavior would increase the behavior, and lead to a larger reduction in burnout, and in burnout covaried factors such as perceived stress, tension, anxiety, and depression, among people with high levels of perceived stress. Of further importance is to investigate how different types of potential recovery behavior, both at work and outside work, in a stress recovery behavior intervention context, mediates changes in burnout in different populations and contexts. Recovery opportunities as a mediator of the change in different recovery behaviors would also be valuable to study.

A brief version of the intervention has been studied using a single-subject experimental design, and studies using such a design control for threats to internal validity [51,52], and systematic replications are necessary to evaluate the external validity [53,54]. For the intervention to be available for as many people interested in taking part in the intervention as possible, this brief version of the intervention warrants further investigation. As continuous measures of potential recovery behaviors and perceptions of recovery opportunities were not used in the testing of the brief version of the intervention, such measures would be of importance to include. Digital versions of the intervention with a low degree of therapist support may also be warranted to study for the sake of availability. Lastly, investigation of physiological measures, such as heart rate variability, which is an established associated factor in burnout [55] and a predictor for both mortality and morbidity [56], possibly accompanied by recovery behaviors, would be of great interest to include when studying functionalistic stress recovery interventions.

## 5. Conclusions

This study shows that the new and innovative functionalistic behavior change intervention BRIGHT, which can be contrasted to interventions with a specific behavioral topographical target (e.g., increasing the degree of time in nature), meaningfully changes its behavioral target: “recovery behaviors” in everyday life. Given that the intervention in a previous study was shown to greatly reduce stress and burnout, it would be valuable to further research the intervention.

## Figures and Tables

**Table 1:** Means and Standard Deviations for Outcome Variables, Differences in Means Between the INT and the WLC at Postassessments with Adjustments for Preassessment values, With 95% CI, and Between-Group Effect Sizes (Cohen’s *d*).

		Pre		Post				
Variable	Measure	*M (SD)*		*M (SD)*		Between-Group		Between-Group
		INT	WLC	INT	WLC	Difference [CI]	*p*	Effect Size [Level]
Perceived recovery opportunities	Control	2.74 (0.99)	2.68 (1.00)	3.41 (0.85)	2.69 (0.82)	0.70 [0.35–1.04]	<0.001	0.75 [high]
Relaxational behavior	Relaxation	2.49 (0.93)	2.55 (1.05)	3.26 (0.84)	2.63 (0.80)	0.64 [0.31–0.98]	<0.001	0.80 [high]
Positively challenging behavior	Mastery	2.28 (0.76)	2.44 (0.84)	2.86 (1.04)	2.85 (0.97)	0.14 [−0.22–0.49]	0.446	0.14 [trivial]

Note. INT = intervention group; WLC = waiting-list control group; ANCOVA = analysis of covariance; CI = Confidence interval. All variables refer only to behaviors during leisure time.

**Table 2:** Clinical Outcomes Regarding the Measures Control, Relaxation, and Mastery at Posttreatment Assessment Separated by Randomized Groups.

	Control		Relaxation		Mastery	
Outcome Category	Post-Treatment, *n* (%)		Post-Treatment, *n* (%)		Post-Treatment, *n* (%)	
	INT (*n* = 35)	WLC (*n* = 38)	INT (*n* = 35)	WLC (*n* = 38)	INT (*n* = 35)	WLC (*n* = 38)
Clinically significant improvement	10 (28.6%)	1 (2.6%)	10 (28.6%)	2 (5.3%)	7 (20.0%)	3 (7.9%)
Reliable improvement	1 (2.9%)	1 (2.6%)	3 (8.6%)	4 (10.5%)	1 (2.9%)	5 (13.2%)
No change	22 (62.9%)	33 (86.9%)	22 (62.9%)	26 (68.4%)	27 (77.14%)	29 (76.3%)
Reliable deterioration	2 (5.7%)	3 (7.9%)	0 (0.0%)	6 (15.8%)	0 (0.0%)	1 (2.6%)

## Data Availability

Data are available from the author upon reasonable request.

## References

[1] McEwen B.S. (2005). Stressed or Stressed out: What Is the Difference?. J. Psychiatry Neurosci..

[2] McEwen B.S. (2006). Protective and Damaging Effects of Stress Mediators: Central Role of the Brain. Dialogues Clin. Neurosci..

[3] Folkman S. (2008). The Case for Positive Emotions in the Stress Process. Anxiety Stress Coping.

[4] Savage D.A., Torgler B. (2012). Nerves of Steel? Stress, Work Performance and Elite Athletes. Appl. Econ..

[5] Eriksen H.R., Murison R., Pensgaard A.M., Ursin H. (2005). Cognitive Activation Theory of Stress (CATS): From Fish Brains to the Olympics. Psychoneuroendocrinology.

[6] De Vente W., van Amsterdam J.G.C., Olff M., Kamphuis J.H., Emmelkamp P.M.G. (2015). Burnout Is Associated with Reduced Parasympathetic Activity and Reduced HPA Axis Responsiveness, Predominantly in Males. Biomed. Res. Int..

[7] Van der Molen H.F., Nieuwenhuijsen K., Frings-Dresen M.H.W., de Groene G. (2020). Work-Related Psychosocial Risk Factors for Stress-Related Mental Disorders: An Updated Systematic Review and Meta-Analysis. BMJ Open.

[8] Almén N., Lisspers J., Öst L.-G. (2020). Stress-Recovery Management: A Pilot Study Using a Single-Subject Experimental Design. Behav. Modif..

[9] Sonnentag S., Venz L., Casper A. (2017). Advances in Recovery Research: What Have We Learned? What Should Be Done Next?. J. Occup. Health Psychol..

[10] Söderström M., Jeding K., Ekstedt M., Perski A., Akerstedt T. (2012). Insufficient Sleep Predicts Clinical Burnout. J. Occup. Health Psychol..

[11] Bosch C., Sonnentag S., Pinck A.S. (2018). What Makes for a Good Break? A Diary Study on Recovery Experiences during Lunch Break. J. Occup. Organ. Psychol..

[12] De Jonge J. (2019). What Makes a Good Work Break? Off-Job and On-Job Recovery as Predictors of Employee Health. Ind. Health.

[13] Sonnentag S., Cheng B.H., Parker S.L. (2022). Recovery from Work: Advancing the Field Toward the Future. Annu. Rev. Organ. Psychol. Organ. Behav..

[14] Lisspers J., Almén N., Sundin Ö. (2014). The Effects of a Recovery-Focused Program for Stress Management in Women—An Exploratory Study. Health.

[15] Skinner E.A., Edge K., Altman J., Sherwood H. (2003). Searching for the Structure of Coping: A Review and Critique of Category Systems for Classifying Ways of Coping. Psychol. Bull..

[16] Bennett A.A., Bakker A.B., Field J.G. (2018). Recovery from Work-Related Effort: A Meta-Analysis. J. Organ. Behav..

[17] Sonnentag S., Fritz C. (2007). The Recovery Experience Questionnaire: Development and Validation of a Measure for Assessing Recuperation and Unwinding from Work. J. Occup. Health Psychol..

[18] Geurts S.A.E., Sonnentag S. (2006). Recovery as an Explanatory Mechanism in the Relation between Acute Stress Reactions and Chronic Health Impairment. Scand. J. Work. Env. Health.

[19] Fritz C., Sonnentag S. (2006). Recovery, Well-Being, and Performance-Related Outcomes: The Role of Workload and Vacation Experiences. J. Appl. Psychol..

[20] Wendsche J., Lohmann-Haislah A. (2017). A Meta-Analysis on Antecedents and Outcomes of Detachment from Work. Front. Psychol..

[21] Chafin S., Christenfeld N., Gerin W. (2008). Improving Cardiovascular Recovery from Stress with Brief Poststress Exercise. Health Psychol..

[22] Almén N., Lundberg H., Sundin Ö., Jansson B. (2018). The Reliability and Factorial Validity of the Swedish Version of the Recovery Experience Questionnaire. Nord. Psychol..

[23] Trógolo M., Morera L., Castellano E.J., Spontón C., Medrano L.A. (2020). Psychometric Properties of the Recovery Experiences Questionnaire in Argentine Workers. Ann. Psicol..

[24] Bennett A.A., Gabriel A.S., Calderwood C., Dahling J.J., Trougakos J.P. (2016). Better Together? Examining Profiles of Employee Recovery Experiences. J. Appl. Psychol..

[25] Hahn V.C., Binnewies C., Sonnentag S., Mojza E.J. (2011). Learning How to Recover from Job Stress: Effects of a Recovery Training Program on Recovery, Recovery-Related Self-Efficacy, and Well-Being. J. Occup. Health Psychol..

[26] Gupta S. (2011). Intention-to-Treat Concept: A Review. Perspect. Clin. Res..

[27] Cohen S., Kamarck T., Mermelstein R. (1983). A Global Measure of Perceived Stress. J. Health Soc. Behav..

[28] Eskin M., Parr D. (1996). Introducing a Swedish Version of an Instrument Measuring Mental Stress.

[29] Almén N., Jansson B. (2021). The Reliability and Factorial Validity of Different Versions of the Shirom-Melamed Burnout Measure/Questionnaire and Normative Data for a General Swedish Sample. Int. J. Stress Manag..

[30] Lundgren-Nilsson Å., Jonsdottir I.H., Pallant J., Ahlborg G. (2012). Internal Construct Validity of the Shirom-Melamed Burnout Questionnaire (SMBQ). BMC Public Health.

[31] Almén N., Sundin Ö., Jansson B. (2022). The Recovery Experience Questionnaire: Normative Data for a General Swedish Sample.

[32] Almén N., Lisspers J., Öst L.-G., Sundin Ö. (2020). Behavioral Stress Recovery Management Intervention for People with High Levels of Perceived Stress: A Randomized Controlled Trial. Int. J. Stress Manag..

[33] Cohen J. (1988). Statistical Power Analysis for the Behavioral Sciences.

[34] Almén N. (2017). Stress—Och Utmattningsproblem: Kognitiva Och Beteendeterapeutiska Metoder.

[35] Sterne J.A.C., White I.R., Carlin J.B., Spratt M., Royston P., Kenward M.G., Wood A.M., Carpenter J.R. (2009). Multiple Imputation for Missing Data in Epidemiological and Clinical Research: Potential and Pitfalls. BMJ.

[36] Sullivan T.R., White I.R., Salter A.B., Ryan P., Lee K.J. (2018). Should Multiple Imputation Be the Method of Choice for Handling Missing Data in Randomized Trials?. Stat. Methods Med. Res..

[37] Babyak M.A. (2004). What You See May Not Be What You Get: A Brief, Nontechnical Introduction to Overfitting in Regression-Type Models. Psychosom. Med..

[38] Harrell F.E. (2015). Regression Modeling Strategies: With Applications to Linear Models, Logistic and Ordinal Regression, and Survival Analysis.

[39] Enders C.K. (2017). Multiple Imputation as a Flexible Tool for Missing Data Handling in Clinical Research. Behav. Res. Ther..

[40] Little R.J.A., Rubin D.B. (2002). Statistical Analysis with Missing Data.

[41] Rubin D.B. (1976). Inference and Missing Data. Biometrika.

[42] Tabachnick B.G., Fidell L.S. (2014). Using Multivariate Statistics.

[43] Rubin D.B. (1987). Multiple Imputation for Nonresponse in Surveys.

[44] Jacobson N.S., Truax P. (1991). Clinical significance: A statistical approach to defining meaningful change in psychotherapy research. J. Consult. Clin. Psychol..

[45] Sonnentag S. (2018). The Recovery Paradox: Portraying the Complex Interplay between Job Stressors, Lack of Recovery, and Poor Well-Being. Res. Organ. Behav..

[46] Rock J.W., Zijlstra F.R.H. (2006). The Contribution of Various Types of Activities to Recovery. Eur. J. Work. Organ. Psychol..

[47] Jacobson N.S., Martell C.R., Dimidjian S. (2001). Behavioral Activation Treatment for Depression: Returning to Contextual Roots. Clin. Psychol. Sci. Pract..

[48] Hill M.N., Hellemans K.G.C., Verma P., Gorzalka B.B., Weinberg J. (2012). Neurobiology of Chronic Mild Stress: Parallels to Major Depression. Neurosci. Biobehav. Rev..

[49] Ottaviani C., Thayer J.F., Verkuil B., Lonigro A., Medea B., Couyoumdjian A., Brosschot J.F. (2016). Physiological Concomitants of Perseverative Cognition: A Systematic Review and Meta-Analysis. Psychol. Bull..

[50] Baraldi A.N., Enders C.K. (2010). An Introduction to Modern Missing Data Analyses. J. Sch. Psychol..

[51] Kazdin A.E. (2011). Single-Case Research Designs: Methods for Clinical and Applied Settings.

[52] Kazdin A. (2017). Research Design in Clinical Psychology.

[53] Barlow D., Nock M., Hersen M. (2009). Single Case Experimental Designs: Strategies for Studying Behavior Change.

[54] Martella C., Nelson R., Marchand-Martella N. (1998). Research Methods: Learning to Become a Critical Research Consumer.

[55] Li J., Zhang M., Loerbroks A., Angerer P., Siegrist J. (2014). Work Stress and the Risk of Recurrent Coronary Heart Disease Events: A Systematic Review and Meta-Analysis. Int. J. Occup. Med. Environ. Health.

[56] Thayer J.F., Lane R.D. (2007). The Role of Vagal Function in the Risk for Cardiovascular Disease and Mortality. Biol. Psychol..

